# Designing Psychological Treatments for Scalability: The PREMIUM Approach

**DOI:** 10.1371/journal.pone.0134189

**Published:** 2015-07-30

**Authors:** Sukumar Vellakkal, Vikram Patel

**Affiliations:** 1 Centre for Control of Chronic Conditions, Public Health Foundation of India, New Delhi, India; 2 Centre for Global Mental Health, London School of Hygiene and Tropical Medicine, London, United Kingdom; 3 Sangath, Goa, India; The George Institute for Global Health, INDIA

## Abstract

**Introduction:**

Lack of access to empirically-supported psychological treatments (EPT) that are contextually appropriate and feasible to deliver by non-specialist health workers (referred to as ‘counsellors’) are major barrier for the treatment of mental health problems in resource poor countries. To address this barrier, the ‘Program for Effective Mental Health Interventions in Under-resourced Health Systems’ (PREMIUM) designed a method for the development of EPT for severe depression and harmful drinking. This was implemented over three years in India. This study assessed the relative usefulness and costs of the five ‘steps’ (Systematic reviews, In-depth interviews, Key informant surveys, Workshops with international experts, and Workshops with local experts) in the first phase of identifying the strategies and theoretical model of the treatment and two ‘steps’ (Case series with specialists, and Case series and pilot trial with counsellors) in the second phase of enhancing the acceptability and feasibility of its delivery by counsellors in PREMIUM with the aim of arriving at a parsimonious set of steps for future investigators to use for developing scalable EPT.

**Data and Methods:**

The study used two sources of data: the usefulness ratings by the investigators and the resource utilization. The usefulness of each of the seven steps was assessed through the ratings by the investigators involved in the development of each of the two EPT, viz. Healthy Activity Program for severe depression and Counselling for Alcohol Problems for harmful drinking. Quantitative responses were elicited to rate the utility (usefulness/influence), followed by open-ended questions for explaining the rankings. The resources used by PREMIUM were computed in terms of time (months) and monetary costs.

**Results:**

The theoretical core of the new treatments were consistent with those of EPT derived from global evidence, viz. Behavioural Activation and Motivational Enhancement for severe depression and harmful drinking respectively, indicating the universal applicability of these theories. All the steps of both phases in PREMIUM contributed to the development of the final EPT. However, if there were significant resource constraints, the steps can be limited to workshops with international and local experts in the first phase, and case series with specialists, and case series and pilot trial with counsellors in the second phase.

**Conclusions:**

Integrating global evidence with local knowledge and practices are complementary and feasible goals to contribute to the development of contextually appropriate and feasible EPT in resource poor country settings. The emerging EPT share similar theoretical frameworks to those described in the global evidence. The PREMIUM method has relevance for any setting where populations have poor access to EPT as its explicit goal is to develop scalable treatments.

## Introduction

About 7.4% of the global burden of disease has been attributed to mental disorders, which rose from 15^th^ leading cause in 1990 to 11^th^ rank in 2010 [1]. The leading causes of this burden are depression in women and harmful alcohol use in men [1]. Empirically-supported psychological treatments (EPT) are amongst the most effective mental healthcare interventions for the treatment of these mental disorders but are not accessible in most populations, in particular low income populations, and most countries, especially in low-and middle income countries [2–6]. This is, in part, due to the very nature of the process of development of EPT which typically begins in highly specialized academic centres, are trialled on patients who are attending mental health clinics (and thus already have a ‘psychological’ explanatory model) and are drawn from a narrow socio-cultural group, and relies on specialized, but scarce and expensive, mental health professionals for delivery [7]. Not surprisingly, disseminating these treatments in the ‘real-world’ of communities and non-specialized healthcare settings, with their diverse populations, many of whom use explanatory models distinct from those of mental health practitioners, and relying on non-specialist health workers for delivery, poses a fresh set of challenges for design and evaluation [8].

PREMIUM, a ‘**Pr**ogram for **E**ffective **M**ental Health **I**nterventions in **U**nder-resourced Health Syste**m**s’, was initiated in India in October 2010 with the explicit goal of designing a method for the development of EPT which are defined by their property of scalability, viz., that they are sensitive to the context of the population in which the EPT will be used, and are feasible for delivery by non-specialist health workers, i.e. health workers who do not have any prior training in mental healthcare. These workers are referred to as ‘counsellors’ in the program to be analogous to other non-specialist health workers who deliver behavioural or ‘talking’ treatments in routine healthcare settings in India. Over the course of three years, PREMIUM implemented this method to develop two EPTs, viz the Healthy Activity Program (HAP) for severe depression [9] and the Counselling for Alcohol Problems (CAP) for harmful drinking [10]. While pilot trials have testified to their acceptability and potential efficacy when delivered in routine primary care settings by counsellors, definitive trials are currently in progress [8].

### The PREMIUM method

The PREMIUM method was guided by a number of *a priori* principles: the treatments had to be based on both global and contextual evidence; they had to be acceptable to populations attending routine healthcare facilities; and they had to be feasible for delivery by non-specialist health workers (in the context of the low resourced setting of the study, this translated to no mental health training of any kind). A core element of the PREMIUM method is a long-term vision of ultimate scalability of EPT by emphasizing their delivery by the same pool of counsellors working in routine primary healthcare settings [8]. Thus, although the two EPT are not trans-diagnostic, they are delivered by the same counsellors in the same healthcare settings concurrently.

**Fig 1:**
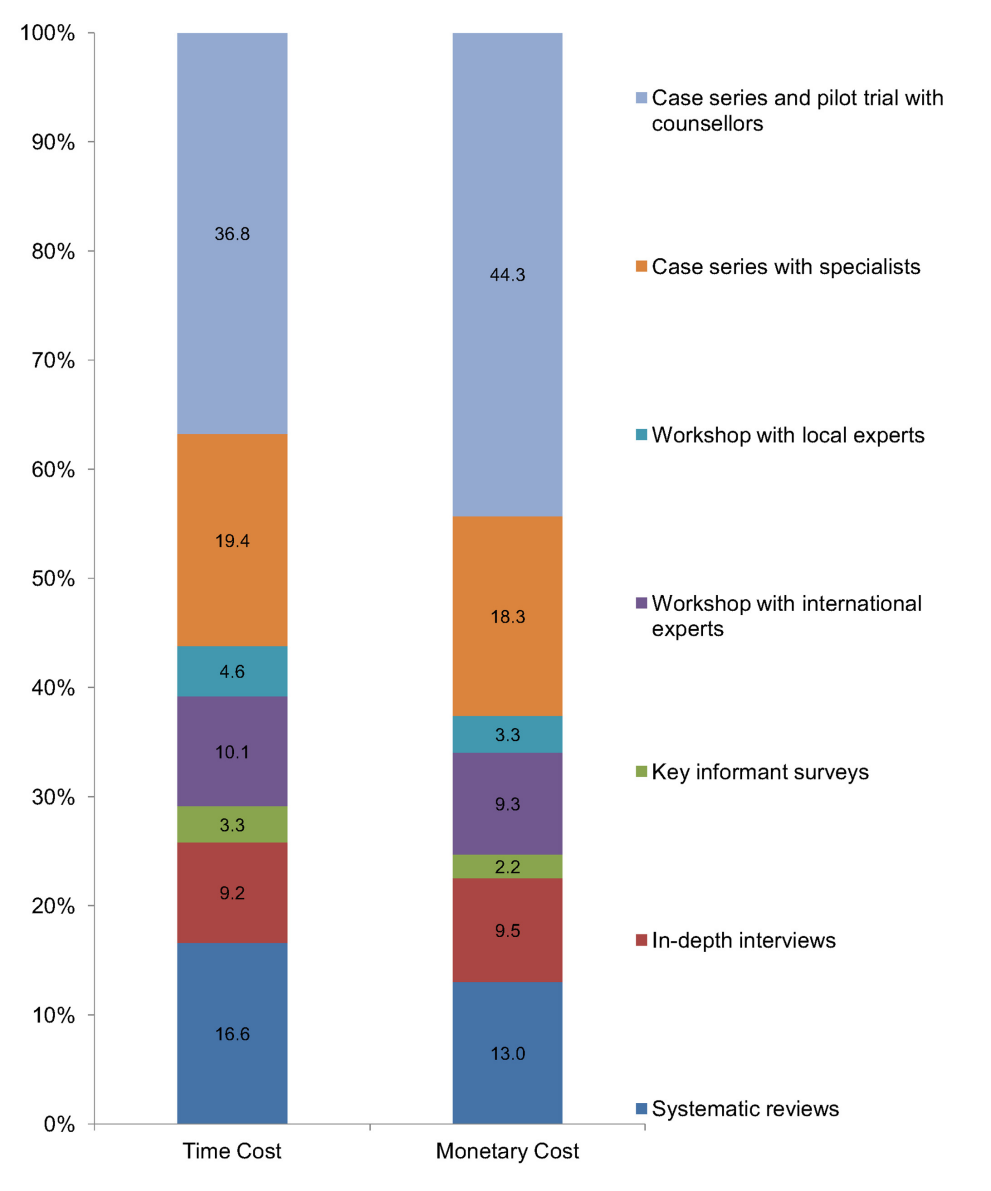
An overview of the steps of PREMIUM method for the development of the scalable EPTs.

The development of the EPT was carried in two broad phases with seven steps (Table 1). The goal of Phase-1 was the identification of strategies and modelling of the delivery and theoretical framework for the treatment. This represents a major deviation from the orthodox model of EPT development in which theory precedes the identification of strategies. Our rationale was that the global EPT were grounded in theories which had already been tested while the contextual ones were grounded in the local population’s historical strategies to address mental health problems. The program involved two Phases with a total of seven steps (Table 1 and Fig 1).

**Table 1:** Phases and steps of the PREMIUM method.

**Phase-1: Identification of strategies and modelling of the treatment**	
Systematic reviews	Synthesised the evidences from the international and regional literatures on EPT strategies as well as the literature on explanatory models of the disorders in south Asia.
In-depth interviews	Conducted with patients, family members and care providers in the PREMIUM study sites to identify the EPT strategies that are used in the local contexts.
Key informant surveys	Conducted with mental health experts and counsellors in India to rate the acceptability, feasibility, effectiveness, and risk of harm in the Indian context for each of the identified EPT strategies that were identified in the reviews and the interviews.
Workshops with international experts	A series of workshops held with international experts to design the psychological treatment based on the strategies identified in the surveys.
Workshops with local experts	A series of workshops held with mental health experts in India in order to design the psychological treatments based on the strategies identified in the surveys and to identify counsellor characteristics.
**Phase-2: Evaluation and refinement of its acceptability and feasibility**	
Case series with specialists	The evaluation of the treatment’s acceptability and feasibility when delivered by mental health experts.
Case series and pilot trial with counsellors	The evaluation of the treatment’s acceptability and feasibility when delivered by counsellors in primary care centres.

The objectives of the analyses reported in this paper were to describe, from the perspectives of the investigators involved with the development of the EPT, the extent of contribution of each step to the goals of the two phases, and to assess the resources used for each of these steps. The ultimate goal was to triangulate these two sets of data to arrive at a parsimonious set of steps which could be used by future investigators seeking to develop scalable EPT in diverse contexts.

## Materials and Methods

All research procedures with human participants were approved by the IRBs of Sangath and the London School of Hygiene & Tropical Medicine, and the Health Ministry Screening Committee of the Indian Council for Medical Research, and written informed consent was obtained from all participants.

The study used two sources of data: the usefulness ratings by the investigators and the resource utilization.

### Assessment of Usefulness

The usefulness of each of the seven steps was assessed through the ratings by the investigators involved in the development of each of the two EPT carried out after the two treatments had been finalized and the definitive trials had started enrolment. A semi-structured questionnaire was piloted with a small group of investigators and the final version was then administered to all investigators through email. To avoid potential biases in responses, the questionnaire was sent to the respondents by the administrative secretary of the Delhi-based research institute who did not know any of the respondents personally. Moreover, the respondents were asked not to leave any personal identifiers in the filled-up questionnaire. Quantitative responses were elicited to rate the following: the utility (usefulness/influence) of each of the steps used in achieving the overall objectives of the respective phases; the likely effectiveness of the EPT in the Indian context; and whether the correct theoretical model had been adopted. Finally, to assess if there is any bias on the ranking on the usefulness of each of the steps due to their differential levels of involvement, the respondents were asked to rank their own influence on the decision making process during the treatment development process. The quantitative rankings were followed by open-ended questions for explaining the rankings on each of these issues.

### Assessment of Resources

The resources used by PREMIUM were computed in terms of time (months) and monetary costs. Costs included both personnel and non-personnel costs such as infrastructure and consumables. The research team asked two senior clinical coordinators, each of whom led one EPT clinical development team in India, to independently estimate the resource use. The following steps were used: the duration of each step was mapped onto the time-chart of the entire project; actual costs for each month were mapped on to this chart; where more than one step occurred in the same month, the approximate proportion of time in that month devoted to each step was estimated; resources (time and costs) were then computed for each step. As the time estimates provided by both the project co-ordinators were similar, and joint consultations were conducted with the co-ordinators to arrive to the final estimates. To account for inflation, the monetary cost was converted in the constant prices of October 2010 using the monthly Wholesale Price Index (WPI) provided by the Government of India [11].

### Analysis

The utility responses were analysed separately for each of the two EPT respondent groups. The transcribed interviews from the qualitative survey among the investigators were read through by the first author to understand the contexts and to identify preliminary themes, and then discussed with the second author. Subsequently, using the grounded theory approach [12], the text data were coded in detail and then inductive method was used to derive major themes. The themes generated were revised after series of discussions between the authors to avoid researcher’s bias and maintain triangulation. The analysis identified which steps were useful in the development of the EPT, and what were the underlying reasons for their usefulness. In addition, the analysis also identified if a combination of steps were found to be useful in the development of the EPT. Descriptive statistics (mean and 95% confidence intervals) was used for analysing the quantitative rankings, and the Spearman’s rank correlation for measuring the association between the ranking on the usefulness and the level of involvement of each of the activities. The utility scores were manually related to the cost data for each step.

## Results

A total of 14 members of the investigators team of the Healthy Activity Program (participation rate 93%) and 10 members of the investigators team of the Counselling for Alcohol Problems (participation rate 91%) completed the survey. Half of the respondents were from India and the rest were foreign. We did not observe any systematic differences between these two groups of respondents in their ratings on the usefulness. Most of the respondents were advisors to the PREMIUM, and had years of research experience in psychological treatment development and evaluation.

### The usefulness of the steps

Overall, the participants rated themselves as having made a considerable influence on the decisions made about the development of the EPT (Table 2). There was no correlation between the level of involvement and the usefulness score (S1 Table).

**Table 2:** Mean (95% CI) of the rating scores of influence, effectiveness and usefulness.

	Healthy Activity Program	Counselling for Alcohol Problems
Influence in the decisions made about the development of the EPT(scale of 1 to 5 where 1 = no influence and 5 = extreme influence)	3.36 [2.73, 3.98]	3.30 [2.40, 4.20]
Effectiveness of the selected treatments(scale of 1 to 5 where 1 = Ineffective and 5 = extremely effective)	3.71 [3.44, 3.98]	3.33 [2.56, 4.10]
Extent the most appropriate theoretical model had been selected(scale of 1 to 5 where 1 = strongly disagree and 5 = Strongly agree)	4.77 [4.50, 5.03]	4.33 [3.79, 4.88]

Table 3 reports the mean scores on the usefulness of each of the seven steps and the rank derived from the usefulness scores. Of the five steps in Phase-1, the workshops with international experts were ranked highest for both EPT, followed by the workshops with local experts and the systematic reviews. Of the two steps of phase-2, the case series and pilot trial with counsellors ranked slightly higher than those with the case series with specialists. Notably, none of the seven steps were rated in the range of scores indicating they were of little or no use.

**Table 3:** Usefulness score and monetary cost rank for the steps in the development of the EPT.

	Healthy Activity Program		Counselling for Alcohol Problems		Monetary Cost
Steps	Rank	Mean (95% CI)	Rank	Mean (95% CI)	Rank
***Phase-1*: *Identification of strategies and modelling of the treatment***					
Workshop with international experts	1	4.75 [4.46, 5.04]	1	4.67 [4.28, 5.05]	3
Workshop with local experts	2	4.64 [4.18, 5.09]	2	4.25 [3.38, 5.12]	2
Systematic reviews	3	4.50 [3.99, 5.01]	3	4.11 [3.14, 5.09]	5
In-depth interviews	4	4.18 [3.68, 4.69]	4	3.67 [2.73, 4.61]	4
Key informant surveys	5	3.91 [3.44, 4.38]	4	3.67 [3.00, 4.33]	1
***Phase-2*: *Evaluation and refinement of acceptability and feasibility***					
Case series and pilot trial with counsellors	1	4.81 [4.59, 5.04]	1	4.93 [4.75,5.10]	2
Case series with specialists	2	4.18 [3.68, 4.69]	2	4.57 [3.84, 5.30]	1

*Notes*: *on the usefulness scale*, *1 = not useful and 5 = extreme useful; on the monetary cost scale*, *a higher rank is assigned for lower relative monetary cost*

The selected EPT were expected to be moderately effective in the Indian context and respondents strongly endorsed the choice of the theoretical model for the two EPT, viz. Behavioural Activation for HAP and Motivational Enhancement for CAP (Table 2).

The major theme emerged from the analyses of the narrative data was the complementarities of each step in the process of the development of the EPT. For instance, the systematic reviews synthesised evidence to identify the interventions which had been found to be effective from the global and local literature, and provided an evidence-based starting point for the EPT development. Subsequently, the in-depth interviews identified contextually relevant and acceptable EPT strategies. The key informant surveys explored the perspectives of healthcare providers on the acceptability, feasibility and potential harm of these strategies if they were delivered by counsellors. This was thus useful to build consensus around strategies, and in shortening the strategy list. Finally, both workshops with international experts and local experts helped to brain-storm about how the various EPT strategies could be delivered in a coherent treatment framework and the theoretical model for the treatment’s effects.

“…all elements were extremely useful and influential, and each contributed to developing our knowledge and understanding of both what the research suggested might work and what local practitioners and patients and family members felt might work and/or be acceptable and appropriate.”

“…each of these stages contributed to selection and short listing of strategies for the treatments based on their effectiveness, cultural relevance and safety when delivered by non-specialists.”

However, if there is less resource at disposal with researchers for developing EPTs, which may be more likely in other contexts (such as those with less time and money than PREMIUM), some respondents suggested for substituting some of steps in phase-1. In particular, the workshops with international experts and local experts could replace the other steps, such as the systematic reviews, in-depth interviews, and key informant surveys.

“…the international experts workshops provided important reflections and many of the important decisions were made during these workshops. The workshops with local experts helped refine the treatment further and also provided validation of the robustness of the evolving treatment… The findings of the intervention development workshops with local experts helped in organizing various strategies into EPT package.”

“…what the systematic review, which has taken more resources, had provided in the process would have arrived at such a consensus in a couple of sittings by the expert/group.

“…opinion surveys (key informant surveys) with experts might be the least valuable as systematic reviews might give the same information. If not enough reviews are available on a given topic, then opinion surveys might be a good substitute.”

On the other hand, both the steps of phase-2 provided direct experience of the acceptability and feasibility barriers that would be encountered in treatment delivery and allowed for important modifications to improve feasibility and acceptability. The involvement of counsellors delivering the treatment in primary care in routine care settings was cited as an invaluable experience in a real world setting.

“…all the steps in phase-2 led to concrete the ground experience of the acceptability of the treatment and strategies and provides basis for formulating the treatment manuals and training the counsellors.”

“…the case series with lay counsellors revealed the very significant barriers in the delivery of the treatment, for e.g. the skills needed to deliver the EPT package, the differing expectations of patients, and the low follow-up rates with primary healthcare based delivery, which we had not anticipated earlier and which greatly influenced our treatment design and delivery plans.”

“the case series with lay counsellors provided important data regarding optimal number of sessions likely to have the most impact on clinical response to treatment”

“…results from phase-2 clearly demonstrate value of process…the case series with specialist and lay counsellors were instrumental in giving operational clarity to the treatment, for e.g. the phases, the need for patient resource materials, the development of quality and competency tools, the barriers to PHC (primary healthcare centre) based delivery etc., which profoundly influenced the structure and delivery of the treatment”

### Resource utilization

**Fig 2:**
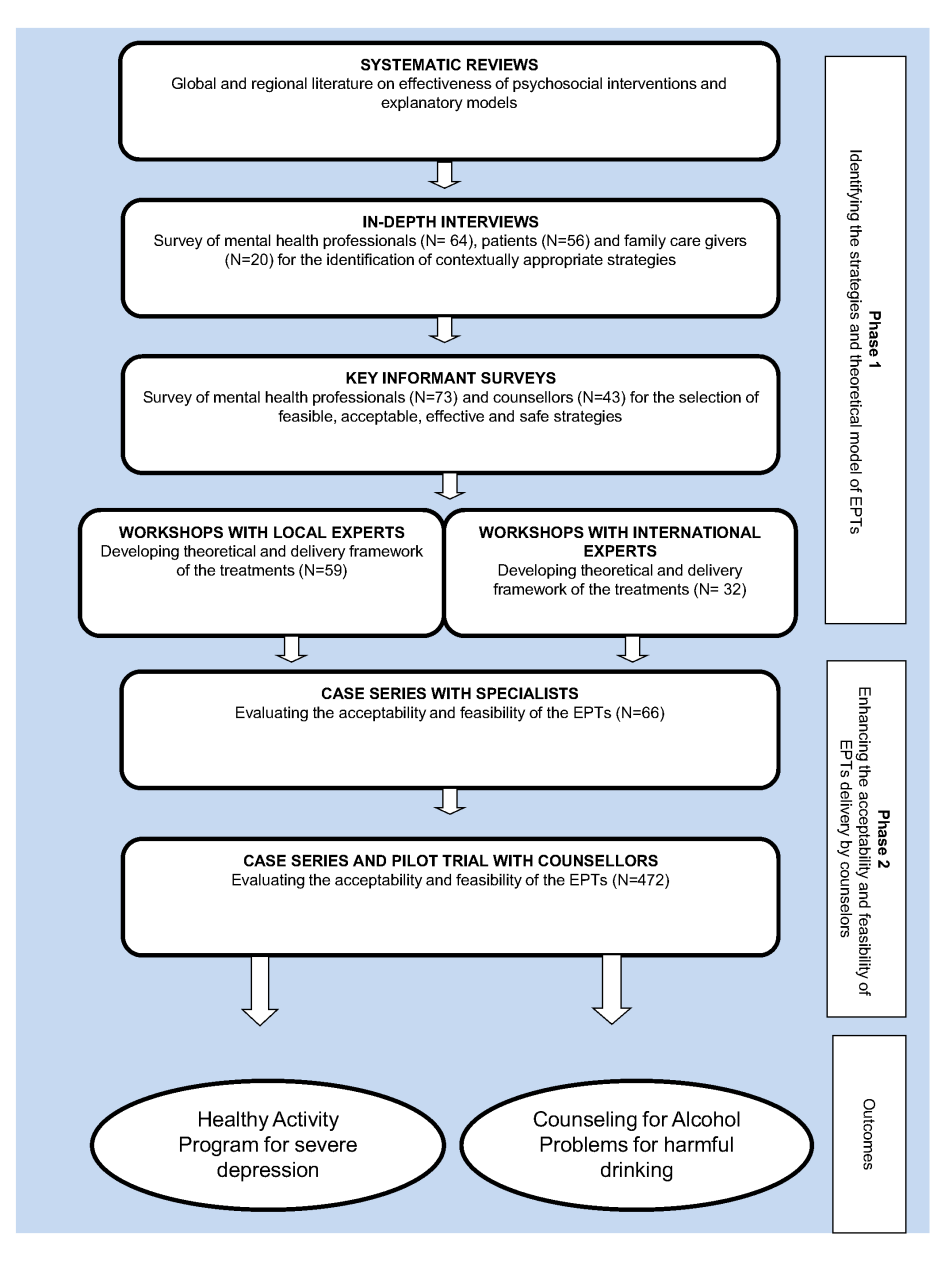
The proportion of time and monetary cost incurred for each step of the development of the PREMIUM treatments.

The PREMIUM method was spread over a 36 month period of time with a total monetary cost of INR 37.04 million (equivalent to US$ 0.57 million). This figure does not include the cost of the Principal Investigator or the time commitment of the international investigators. As shown in Fig 2, phase-2 accounted for just over half of the total time and monetary cost of which the case series and pilot trial with counsellors accounted for over 65.4% and 70.8% of the monetary and time cost respectively. Amongst the steps in phase-1, the systematic reviews was the most expensive and consumed about 37.9% and of the time and 34.8% of the monetary cost while the key informant surveys was the least-expensive (7.6% of the time and 6.0% of the monetary cost).

### Comparison of the usefulness and resource utilization

A comparative assessment of the rankings based on the usefulness and resource cost for each step (Table 3) show a mixed pattern. For phase-2, the higher usefulness scores were positively related to the higher levels of resource utilization. However, an inverse association was found for steps of the phase-1. Thus, the workshops with international experts and local experts were ranked first and second on the usefulness score. These steps used relatively less resources than the systematic reviews and in-depth interviews which had lower usefulness ranks but consumed substantial resources. Moreover, the key informant surveys was ranked lowest in terms of usefulness and was also the least expensive step.

## Discussion

This study evaluated the usefulness of and resources required for each of the seven steps in two phases of the PREMIUM method with the goal of developing scalable EPT for the two leading mental health contributors to the global burden of disease. The study found that all the steps significantly contributed to the final treatment and were complementary to each other while developing the EPT. Notably, our approach grounded in both global and contextual evidence ultimately led to EPT whose theoretical core were consistent with those of widely used EPT derived from global evidence, viz. Behavioural Activation and Motivational Enhancement, indicating the universal applicability of these theories. This observation strengthens the confidence with which such psychological theories can be generalized across cultural contexts, a finding consistent with the experiences of other investigators seeking to adapt psychological treatments for use in culturally diverse settings [13].

Our study could inform future researchers on the methods for the development of scalable EPT in culturally diverse populations. If resources permit, we suggest applying all the steps used in PREMIUM for developing EPT which integrate global evidence with local knowledge and practices, and are contextually appropriate, and can be delivered by non-specialist health workers. On the other hand, if there are resource constraints, only the most important steps can be applied. In particular, the workshops with international experts and local experts in phase-1, and the case series with specialists, and case series and pilot trial with counsellors in phase-2 form the essential steps. As the international and local experts are familiar with the evidence base regarding EPT and local experts are additionally familiar with the contextual barriers to the delivery of EPT by counsellors, the workshops with international and local experts could replace both the systematic reviews and the key informant surveys. Similarly, the key informant surveys that had explored the perspectives of healthcare providers on the acceptability, feasibility and potential harm of these strategies if they were delivered by counsellors can be replaced by the workshop with local experts who are familiar with the locally relevant EPT. Furthermore, since the rankings of usefulness of the seven steps do not vary greatly, our final conclusion was based on the findings from the qualitative responses on the value addition of each step for the final EPT, the costs incurred for each step, and potential resource constraints in the study setting. However, there is a risk of losing some important contributions when removing these steps from the process of EPT development. Thus, one could strike a compromise by reducing the intensity of the step so that its contribution is still maintained while still reducing its resource utilization, for example by replacing systematic reviews with a rapid non-systematic reviews or high quality guidelines.

Our study has several limitations. First, our estimation of costs only captured the direct costs attributable to the grant and thus did not include the indirect costs (such as the time of the international investigators which were not billed to the grant) or that of the PI (who was part funded by the grant and holds an appointment with a UK university). However, even if the PI cost was included, it would not materially affect the relative ranking of the costing of each step as this is based on proportions of time spent on each step. Second, the resource cost can vary in different contexts, limiting the generalizability of our findings, a primary reason why we put less emphasis on absolute amounts. Finally, a larger sample size of respondents may have yielded more accurate estimates on the usefulness of each of the steps. However, we were constrained with the available number of respondents who were required to be familiar with the PREMIUM methodology. We had very high participation rate of the PREMIUM investigators in our survey.

The method used by PREMIUM follows a framework which is similar to the methods used by other investigators to successfully adapt EPT for use in diverse cultural contexts [13–15] indicating the generalizability of these methods across applications and settings. This study implies that integrating global evidence with local knowledge and practices is a feasible goal to contribute to the development of locally relevant and feasible EPT in resource poor country settings. In all these adaptations, the underlying frameworks is that of the development and evaluation of complex public health interventions such as the one outlined by the Medical Research Council of the UK [16]. There is now robust evidence that counsellors can be trained to deliver EPT effectively for people with depressive and substance-use disorders in low- and middle-income countries [17]. The PREMIUM method has been adapted for the development of two brief structured treatments for severe depression and harmful drinking in India, for delivery by non-specialist health workers in routine primary care or community based settings. Although there are several EPT for a wide range of mental disorders, and indeed for many other health conditions, the vast majority of people who can benefit from these treatments do not receive them [3, 18, 19]. Major barriers to the access to EPT in countries like India include lack of specialist human resources to deliver these treatments, stigma, economic barriers and different explanatory models [18, 20]. Disseminating the EPT in these countries requires their contextual acceptability and feasibility for delivery by non-specialist workers where most of the available EPT were developed in ‘western’ cultural, clinical and academic settings [7, 20]. The PREMIUM method has relevance for any setting where populations have poor access to EPT as its explicit goal is to develop scalable treatments.

## Supporting Information

S1 Table Correlation between level of involvement and usefulness ratings for each method. (PDF)
