# Correction: Designing Psychological Treatments for Scalability: The PREMIUM Approach

**DOI:** 10.1371/journal.pone.0136976

**Published:** 2015-08-25

**Authors:** The PLOS ONE Staff

The images for Figs 1 and 2 are incorrectly switched. The image that appears as Fig 1 should be Fig 2, and the image that appears as Fig 2 should be Fig 1. The figure captions appear in the correct order. The publisher apologizes for the error.
