# Supplementary material for: Designing Psychological Treatments for Scalability: The PREMIUM Approach
Source: PLoS One. 2015 Jul 30;10(7):e0134189. doi: 10.1371/journal.pone.0134189 (PMC4520585; doi:10.1371/journal.pone.0134189)
Supplement: S1 Table — (PDF) [file pone.0134189.s001.pdf]

S1 Table. Correlation between level of involvement and usefulness ratings for each method

		Correlation coefficient	P value	N
1	Systematic reviews	-0.07	*0.76*	20
2	In-depth interviews	-0.01	*0.97*	20
3	Key informant surveys	-0.48	*0.03*	20
4	Workshop with international experts	0.03	*0.91*	21
5	Workshop with local experts	0.20	*0.42*	18
6	Case series with specialists	0.47	*0.05*	18
7	Case series and pilot trial with counsellors	-0.17	*0.50*	18
